# Fasinumab for osteoarthritis: a systematic review and meta-analysis of efficacy and safety

**DOI:** 10.1186/s12891-026-10271-8

**Published:** 2026-07-24

**Authors:** Muhammad Sohaib Khan, Wania Moeen, Umm E Salma Shabbar Banatwala, Muhammad Usman, Umar Mahmood, Syed Ashad Ahmed Fatmi, Syed Muhammad Muneeb Akhtar, Ashir Kanwal, Hania Shahzad, Muhammad Talal Ibrahim, Badaruddin Sahito, Muhammad Sohaib Asghar

**Affiliations:** 1 Department of Medicine, Dow University of Health Sciences, Karachi, Pakistan; 2 Liaquat University of Medical and Health Sciences, Jamshoro, Pakistan; 3 UC Davis Health, Sacramento, California U.S.A.; 4 Department of Orthopaedics, The Ohio State University Wexner Medical Center, Columbus, Ohio U.S.A.; 5 Department of Orthopaedic Surgery, Dow University of Health Sciences, Karachi, Pakistan; 6 Department of Internal Medicine, University of Florida (UF Health), UF Spanish Plaines Hospital, The Villages, FL USA; 7 AdventHealth Sebring, Sebring, FL USA

**Keywords:** Fasinumab, Osteoarthritis, Safety, Efficacy, Meta-analysis, MCID

## Abstract

**Objective:**

Fasinumab, a newly developed anti-nerve growth factor monoclonal antibody, has been evaluated in clinical trials for its potential to alleviate symptoms in osteoarthritis patients. This meta-analysis evaluates the efficacy and safety of different Fasinumab dosages compared with placebo in patients with osteoarthritis.

**Methods:**

PubMed (MEDLINE), Cochrane CENTRAL, ClinicalTrials.gov, and Google Scholar were searched for double-blinded RCTs comparing Fasinumab with placebo till December 2025. Primary outcomes were WOMAC pain, WOMAC function, and Patient Global Assessment (PGA) scores. Secondary outcomes included safety profile such as Adjudicated Arthropathy, Adverse events, serious adverse events, all-cause mortality. Cochrane Risk of Bias 2 was used for quality assessment. A random effects model was used for analysis, with subgrouping by dosage.

**Results:**

Six RCTs, involving 9,429 participants, were included. All trials included had a low risk of bias. At 16 weeks, 1 mg Subcutaneous (SC) Every four weeks (Q4W), 1 mg SC Every eight weeks (Q8W), and 3 mg SC Q4W showed a greater statistically significant reduction in WOMAC pain and function scores compared to placebo. However, according to minimal clinically important differences (MCIDs), only 1 mg SC Q4W and 3 mg SC Q4W may provide clinically meaningful reductions in WOMAC pain ([MD: -1.05, (95% C.I. -1.32 to -0.78), *p* < 0.00001, I²=28%] and [MD: -1.16, (95% C.I. -1.58 to -0.75), *p* < 0.00001, I²=0%], respectively) and function scores ([-1.05 (95% C.I. -1.32 to -0.79), *p* < 0.00001, I²=20%] and [MD: -1.24, (95% C.I. -1.65 to -0.83), *p* < 0.00001, I²=0%], respectively) more than placebo; however, the confidence intervals include effects smaller than the MCID threshold, limiting certainty regarding clinically meaningful benefit. A 1 mg SC Q4W dosage regimen is favored over a placebo in improving PGA scores at 16 weeks. Further, Fasinumab use may increase the risk of adverse events, including Joint-replacement surgery and Adjudicated Arthropathy.

**Conclusion:**

Although select fasinumab regimens demonstrated statistically significant reductions in WOMAC pain, function, and PGA scores at 16 weeks, confidence interval overlaps with conservative MCID thresholds limit certainty regarding clinically meaningful efficacy. Additionally, treatment may carry increased risks, including adjudicated arthropathy.In addition, osteoarthritis is a chronic disease, and these short-term efficacy outcomes may not reflect long-term treatment effectiveness. These findings are based on trials assessed as having an overall low risk of bias. Further research is needed to confirm these findings.

**Supplementary Information:**

The online version contains supplementary material available at https://doi.org/10.1186/s12891-026-10271-8.

## Introduction

Osteoarthritis (OA), a chronic and degenerative condition, arises from damage to the joint cartilage resulting in pain, swelling, stiffness, and loss of function [1, 2]. This debilitating condition is recognized as a global endemic, impacting nearly 7% of the world’s population [3]. In the United States alone, where almost 30 million individuals are affected by OA, with associated healthcare expenditures exceeding $130 billion annually [4, 5]. A substantial proportion of this economic burden is attributable to the management of OA-related pain [6].

Joint pain in OA is one of the principal reasons for seeking medical assistance [7]. This pain can be driven by both nociceptive and neuropathic means [8]. Numerous pathways are linked with OA pain including nerve growth factor, calcitonin gene-related peptide, tumor necrosis factor-α, and C-C motif chemokine ligands 2, and arachidonic acid–derived lipid mediators such as prostaglandins [7]. Strategies for pain management encompass nerve growth factor inhibitors, platelet rich plasma (PRP), tumor necrosis factor antibodies, interleukin-1β, natural growth factors, and ion channel modulators, however these treatment are experimental [9–11].

Anti-nerve growth factor antibodies such as tanezumab when administered intravenously have shown improvements in joint function and pain relief amongst patients with severe hip or knee OA, however, it may result in rapid progression of OA [12]. Adalimumab, a tumor necrosis factor antibody, has shown significant improvements in pain scores but may increase the risk of serious infections such as reactivation of latent tuberculosis [13, 14]. Other medications such as nonsteroidal anti-inflammatory drugs and cyclooxygenase inhibitors are recommended as first-line treatments due to their efficacy but are associated with heightened cardiovascular risks [15]. Intra-articular corticosteroid injections are commonly employed for short term improvements in pain and functional outcomes in OA patients but come with potential side effects, such as accelerated OA progression, subchondral insufficiency fracture, and complications of osteonecrosis [16].

Another medication, Fasinumab, an anti-nerve growth factor monoclonal antibody, has been shown to reduce pain in OA patients [17]. This immunoglobulin G4 hinders the signaling of nerve growth factor through tropomyosin receptor kinase A and low-affinity p75 neurotrophin receptors [18, 19]. Clinical trials have already evaluated the efficacy of Fasinumab for OA and found it to provide significant benefits over placebo [20]. However, Fasinumab’s use is also suspected to increase unanticipated joint deterioration and sympathetic nervous system dysfunction [20]. Currently, to the best of our knowledge, no systematic review and meta-analysis addressing Fasinumab’s efficacy and safety exists in literature. Additionally, existing trials have not sufficiently elucidated the cumulative effects of specific doses. Therefore, our study aims to fill these gaps by analyzing and comparing the efficacy and safety profiles of different Fasinumab dosages against placebo in individuals with OA.

## Materials and methods

This systematic review and meta-analysis was performed in accordance with the guidelines of the Preferred Reporting Items for Systematic Reviews and Meta-analysis (PRISMA) statement and was registered in PROSPERO (CRD42023480175) [21].

### Inclusion and exclusion criteria

In the exploration of Fasinumab’s efficacy and safety versus placebo, our inclusion criteria were designed to encompass: (1) double-blinded randomized control trials (RCTs) or better; (2) the thoroughness of dual-arm studies or better, presenting comprehensive data for both Fasinumab and placebo or Normal Saline (NS); (3) the inclusion of patients with radiographically confirmed OA ((Kellgren-Lawrence) KL grade > = 2); and (4) Fasinumab administration solely through subcutaneous injections. Only English language and human studies were selected in the search.

### Data sources and searches

A systematic literature search of PubMed (MEDLINE), Cochrane CENTRAL, ClinicalTrials.gov, and Google Scholar were conducted for relevant studies from inception up to Deccember 2025. The following terms were used: Fasinumab, Osteoarthritis, and REGN 475. MeSH terms were used where possible. The complete search strategy is available in Supplementary Table 1. The references of each of the retrieved studies were examined to identify other studies that conformed to our eligibility criteria.

### Data extraction

Two authors (U.M. and M.S.K.) independently reviewed titles and abstracts of the studies identified by searches, removed duplicates, and performed study selection based on our eligibility criteria. Data extraction was performed independently by one author (U.S.B.) and cross-checked by a second author (M.U.), who also performed independent extraction. Any discrepancies between the two authors were resolved through discussion. Sample size, study duration, study type, mean body mass index (BMI), mean age, treatment period, trial number, key elements for risk of bias assessment, publication year, location, inclusion and exclusion criteria for individual studies, female (%), location of OA, patient population and all relevant outcomes were extracted. Relevant outcomes included Western Ontario and McMaster Universities Osteoarthritis Index (WOMAC) pain, WOMAC function score and Patient Global Assessment scores (PGA), all reported at 16 weeks, which were also the primary outcomes. The 16-week timepoint was selected because it was the most consistently reported primary endpoint across the included studies.All-Cause Joint Replacement, Adjudicated Arthropathy, Adverse Events, Serious Adverse Events, Treatment Emergent Adverse Events (TEAEs), Sympathetic Nervous System Dysfunction, Peripheral Sensory Adverse Events, Peripheral Edema, Rapidly Progressing Osteoarthritis (RPOA) and All-Cause Mortality were also extracted from our included studies; they were made the secondary outcomes. Definition of outcomes are provided in Supplementary Table 2 where applicable. Any disputes in any of the processes were resolved by another investigator (W.M).

### Quality assessment and publication risk of bias

In assessing study quality, we utilized the Cochrane-recommended ROB 2 tool (Supplementary Fig. 1) [22]. Five domains were separately evaluated: randomization process, deviations from intended interventions, missing outcome data, measurement of the outcome, and selection of the reported result. Two independent reviewers (M.T.I and H.S) analyzed the included studies, with discrepancies resolved through discussion with a third reviewer (B.S). Categorization of risk of bias as low, high, or some concern was based on judgments across these domains. Studies demonstrating low bias were considered internally valid, whereas those exhibiting high or unclear bias were approached cautiously, potentially leading to their exclusion from the analysis. This meticulous approach secured a thorough examination of study quality in our meta-analysis, ensuring a comprehensive evaluation. Publication bias assessment was not performed as the total number of included studies is less than 10, which is the criterion.

### Data analysis

Review Manager, version 5.4.1 (Copenhagen: The Nordic Cochrane Centre, The Cochrane Collaboration, 2014) was used for the meta-analysis of the pooled dataThe I^2^ statistics were used to assess the heterogeneity by visual evaluation of forest plots. A I^2^ value exceeding 75% was deemed high heterogeneity [23]. A sensitivity analysis was conducted whenever the heterogeneity was discovered as high. A Baujat plot was also utilized to examine patterns of heterogeneity by demonstrating the relationship between the influence of effect size and the contribution to heterogeneity (Supplementary Fig. 2–3). On the x-axis of the plot, individual studies’ contributions to the overall heterogeneity statistics are represented, while the y-axis illustrates the overall treatment effect with and without each study. This measure on the y-axis offers insights into the influence of each study on the overall treatment effect. P value < 0.05 was statistically significant. When dichotomous data was reported as the number of cases or events instead of the number of patients involved, that data was not included in the forest plots. A minimum of data from 2 different studies was required for the synthesis of a forest plot. In cases where dichotomous data was reported as 0 events in both the intervention and placebo arms, that data was still considered insightful and all concerned forest plots were created; such studies were automatically excluded from the pooled estimate by RevMan using the Mantel-Haenszel method (displayed as ‘Not Estimable’), and no continuity correction was applied. We refined the accuracy of our results by stratifying them according to the different dosages of Fasinumab. For trials with multiple intervention arms sharing a common placebo group, each dose comparison was analyzed as a separate subgroup, with no overall pooled estimate generated combining these subgroups, thereby avoiding double-counting of the shared control arm. The MD and 95% confidence intervals were pooled using the random effects model for the continuous outcomes. Risk Ratio (RR) using the Mantel-Haenszel method was used for dichotomous outcomes. We also conducted an analysis to assess the differences in efficacy among the different drug regimens reported in the studies. Using Review Manager’s “Test for Subgroup Differences” functionality, we evaluated whether these distinctions achieved statistical significance or not. For the WOMAC pain score and WOMAC function scores, the pooled effect sizes were compared with their Minimum Clinically Important Differences (MCID)—defined as the minimum change in a Patient Reported Outcome Measure (PROM) that would have a clinically meaningful effect. When the magnitude of the treatment effect equals or exceeds the MCID, the patient will clinically feel the presence of the treatment. Based on previous work, MCID values for WOMAC pain scores (scale of 0–10) range from 0.75–0.97, while MCID values for WOMAC function scores (scale of 0–10) were found to range between 0.67–0.93 [24, 25].

## Results

### Study selection

**Fig. 1:**
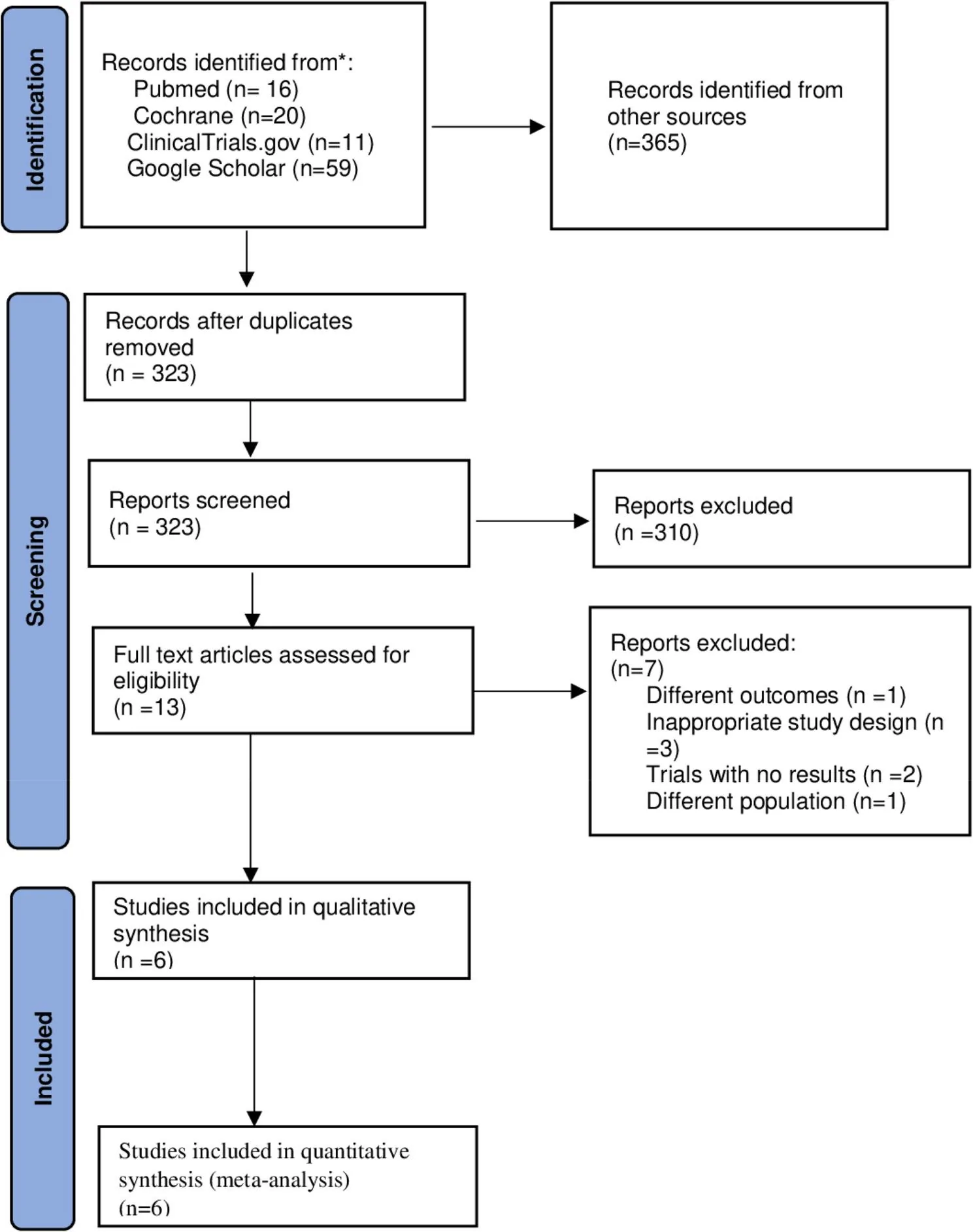
Preferred Reporting Items for Systematic Reviews and Meta-analyses (PRISMA) flow diagram for identifying and selecting studies

From a total of 471 results obtained from the literature search, 323 articles were left after duplicate removal. From these 323 articles, 310 were excluded based on title and abstract review and 13 were selected for full text screening. From the 13, seven studies were removed due to reasons including, different outcome (*n* = 1), inappropriate study design (*n* = 3), trials reporting no results (studies for which no outcome data were publicly available, either because the results were not reported on ClinicalTrials.gov or the publication contained no results) (*n* = 2) and different patient population (*n* = 1) Finally, six randomized control trials including 9,429 participants met the eligibility criteria and were included in our study [25–30] **(**Fig. 1).

### Study and patient characteristics

The study characteristics and patient characteristics are reported in Tables 1 and 2 respectively. BMI data were reported in two trials, with mean values ranging from 24.8 to 31.8 kg/m² across Fasinumab dosage groups [25, 29]. Obesity according to the World Health Organization (WHO) classifications is defined with BMI equal or greater than 30.0 kg/m² [31]. The remaining four trials did not report BMI data [26–28, 30].

**Table 1:** Study characteristics

Trial number	Year	Location	Patient Inclusion	Patient Exclusion	Treatment period	Dosages involved	Outcomes of interest	Study type	Total participants
Dakin P et al. (NCT02447276) [26]	2019	United States	BMI ≤ 39; OA pain history; moderate–severe joint pain; regular NSAID/opioid use; willing to discontinue treatments.	Patients with inflammatory joint disease, osteonecrosis, joint trauma, fibromyalgia, joint replacement, subchondral fracture, HA/corticosteroid injections, neuropathies, pregnancy/breastfeeding; patients with no pregnancy test result	12 weeks	Fas 1 mg SC Q4W Fas 3 mg SC Q4W Fas 6 mg SC Q4W Fas 9 mg SC Q4W	WOMAC pain subscale score; WOMAC physical function subscale score; PGA score; TEAEs; AE; RPOA; AA; All-Cause JRS;	Triple blinded RCT	Fas 1 mg: 85 Fas 3 mg: 84 Fas 6 mg: 85 Fas 9 mg: 84 Placebo: 83
NCT02683239 [27]	2021	Bulgaria, Chile, Colombia, Denmark, Estonia, Germany, Hong Kong, Hungary, Italy, Lithuania, Mexico, Peru, Poland, Romania, Russian Federation, South Africa, Spain, Sweden, Ukraine, United Kingdom, United States	Moderate–severe knee/hip OA; WOMAC ≥ 4; ≥12 wk analgesic use; regular medication use.	Patients with non-OA inflammatory joint disease, arthropathy, CTS, MRI candidates, planned joint replacement, systemic steroids, MS, autonomic neuropathy/failure, pregnancy/breastfeeding, childbearing potential	52 weeks	Fas 1 mg SC Q8W Fas 1 mg SC Q4W Fas 3 mg SC Q4W Fas 6 mg SC Q8W Fas 6 mg SC Q4W Fas 9 mg SC Q4W	TEAEs; AE; SAE; AA; All-cause JRS; WOMAC Pain and Physical Function Subscale Score; PGA; ACM; Sympathetic Nervous System Dysfunction: Peripheral Sensory Adverse Events. RPOA; Peripheral Edema	Quadruple blinded RCT	Fas 1 mg Q8W: 971 Fas 1 mg Q4W: 975 Fas 3 mg Q4W: 214 Fas 6 mg Q8W: 1680 Fas 6 mg Q4W: 116 Fas 9 mg Q4W: 115 Placebo: 1260.
NCT03304379 [28]	2020	United States	Willing to discontinue pain meds/supplements; follow rescue treatment and ≥ 12-wk inadequate pain relief or analgesic intolerance.”	Patients with autoimmune/inflammatory/traumatic joint pathology, prior corticosteroid or anti-NGF exposure, sensitivity to antibodies, and childbearing potential not using contraception.	24 weeks	Fas 1 mg SC Q4W Fas 3 mg SC Q4W Fas: 6 mg SC Q8W	AA; TEAE; All-cause JRS; AE and SAEs; ACM; Sympathetic Nervous System Dysfunction; Peripheral Sensory Adverse Events; RPOA	Double blinded RCT	Fas 1 mg: 612 Fas 3 mg: 59 Fas 6 mg: 59 Placebo: 308
NCT03161093 [29]	2021	Denmark, Germany, Hungary, Lithuania, Poland, Romania, Russian Federation, South Africa, Spain, Ukraine, United Kingdom, United States	Moderate–severe OA pain; discontinued pain meds/supplements; used rescue treatment; radiograph consent; maintained activity & follow-up; Participants in the Year 2 of study: ≥10 prior doses + consent.”	Patients with non-OA inflammatory joint disease, arthropathy, trauma, CTS, autonomic failure, naproxen intolerance, arrhythmias/orthostatic hypotension, uncontrolled HTN, corticosteroid/anti-NGF exposure, and NRS non-compliance.	44 weeks	Fas 1 mg SC Q8W Fas 1 mg SC Q4W Fas 3 mg SC Q4W Fas 6 mg SC Q8W	WOMAC Pain Subscale Score; PGA score; TEAEs; WOMAC Physical Function Subscale; AA; All cause JRS; AE; SAE; ACM; Sympathetic Nervous System Dysfunction; Peripheral Sensory; AE; RPOA; Peripheral Edema.	Triple blinded RCT	Fas 1 mg: Q8W 551 Fas 1 mg Q4W: 1054 Fas 3 mg Q4W: 145 Fas 6 mg Q8W: 140 Placebo: 354
NCT03245008 [30]	2021	Japan	Moderate–severe OA pain; WOMAC ≥ 4; inadequate NSAID/opioid relief; BMI ≤ 39; lifestyle compliance; post-op follow-up; able to complete endpoints.	Patients with CTS, inflammatory arthropathy, autonomic neuropathy/familial dysautonomia, naproxen intolerance, systemic steroids, HA injections, arrhythmias/orthostatic hypotension, NRS non-compliance, pregnancy/breastfeeding, prior anti-NGF use.	44 weeks	Fas 1 mg SC Q4W Fas 1 mg SC Q8W Fas 3 mg SC Q4W Fas 6 mg SC Q8W	AE; SAE; TEAE ; WOMAC Pain and Physical Function Subscale Score; PGA Score; Participants with JRS; ACM; RPOA	Quadruple blinded RCT	Fas 1 mg Q4W: 203 Fas 1 mg Q8W: 162 Fas 3 mg Q4W: 28 Fas 6 mg Q8W: 29 Placebo: 204
NCT03691974 [31]	2021	United States, Poland, United Kingdom	Moderate-to-severe joint pain; regular use of analgesic medications; consent	Patients with non-OA inflammatory joint disease, Paget’s disease, neuropathic disorders, MS, fibromyalgia, tumors, renal osteodystrophy, joint trauma, compression neuropathy, uncontrolled diabetes, HIV/AIDS, HTN, hepatitis B/C.	12 weeks	Fas 1 mg SC Q4W	WOMAC Pain and Physical Function Subscale; TEAEs; AA Events; All-cause JRS; ACM; AE; SAE; Sympathetic Nervous System Dysfunction; RPOA	Quadruple blinded RCT	Fas 1 mg: 91 Placebo: 89.

*JRS* Joint Replacement Surgery, *SC* Subcutaneous, *AA* Adjudicated Arthropathy, *WOMAC* Western Ontario and McMaster Universities Osteoarthritis Index, *RCT* Randomized Controlled Trial, *PGA* Patient Global Assessment, *AE* Adverse Events, *NSAIDs* Non-steroidal Anti-Inflammatory Drugs, *BMI* Body Mass Index (kg/m³), *Q4W* Every four weeks, *Q8W* Every eight weeks, *mg* Milligrams, *Fas* Fasinumab, *OA* Osteoarthritis, *TEAEs* Treatment Emergent Adverse Events, *ACM* All Cause Mortality, *HIV* Human Immunodeficiency Virus, *AIDS* Acquired Immunodeficiency Syndrome, *MRI* Magnetic Resonance Imaging, *NGF* Nerve Growth Factors, *NRS* Numeric Rating Scale, *SAEs* Serious Adverse Events, *CTS* Carpal Tunnel Syndrome, *wk* week

**Table 2:** Baseline characteristics

Trial number/ Study Author	Age; mean (SD)		BMI; mean (SD)		Female; %		Patient population	Osteoarthritis location
	Fas	Placebo	Fas	Placebo	Fas	Placebo		
Dakin P et al. (NCT02447276)* [26]	Fas 1 mg: 60.7 (8.9) Fas 3 mg: 60.7 (8.9) Fas 6 mg: 60.1 (7.9) Fas 9 mg: 61.5 (7.8)	60.1 (7.2)	Fas 1 mg: 30.6 (5.0) Fas 3 mg: 30.9 (4.7) Fas 6 mg: 30.5 (4.9) Fas 9 mg: 31.8 (5.0)	31.8 (4.5)	Fas 1 mg: 69.4% Fas 3 mg: 64.3% Fas 6 mg: 60% Fas 9 mg: 64.3%	65.1%	Adults 40–80 year; ACR OA criteria; K–L ≥ 2; knee/hip OA.	Fas 1 mg: Knee (75) or Hip (10) Fas 3 mg: Knee (74) or Hip (10) Fas 6 mg: Knee (74) or Hip (11) Fas 9 mg Q4W: Knee (74) or Hip (10) Placebo: Knee (74) or Hip (9)
NCT02683239 [27]	Fas 1 mg Q8W: 65.0 (9.24) Fas 1 mg Q4W: 65.1 (8.57), Fas 3 mg Q4W: 65.3 (9.19) Fas 6 mg Q8W: 62.9 (9.61) Fas 6 mg Q4W: 62.4 (8.38) Fas 9 mg Q4W: 63.4 (9.89)	63.7 (9.24)	NA	NA	Fas 1 mg Q8W: 74.5% Fas 1 mg Q4W: 72.3% Fas 3 mg Q4W: 69.6% Fas 6 mg Q8W: 73.6% Fas 6 mg Q4W: 71.6% Fas 9 mg Q4W: 66.1%.	72.5%	Adults ≥ 18 year; ACR OA criteria; K–L ≥ 2; knee/hip OA.	Knee or Hip
NCT03304379 [28]	Fas 1 mg Q4W: 62.1 (9.13) Fas 3 mg Q4W: 62.8 (9.38) Fas 6 mg Q8W: 61.5 (9.55)	62.0 (9.30)	NA	NA	Fas 1 mg Q4W: 71.2% Fas 3 mg Q4W: 61% Fas 6 mg Q8W: 57.6%;	72.1%	Adults ≥ 18 year; ACR OA criteria; K–L ≥ 2; knee/hip OA	Fas 1 mg Q4W: Knee (554) or Hip (58)
NCT03161093 [29]	NA	NA	NA	NA	Fas 1 mg Q8W: 76% Fas 1 mg Q4W: 75.2% Fas 3 mg Q4W: 66.2% Fas 6 mg Q8W: 70%	74.9%	Adults ≥ 18 year; ACR OA criteria; K–L ≥ 2; knee/hip OA.	Fas 1 mg Q4W: Knee (893) or Hip (159) Fas 1 mg Q8W: Knee (469) or Hip (85) Placebo: Knee (302) or Hip (50)
NCT03245008 [30]	Fas 1 mg Q4W: 66.1 (9.7) Fas 1 mg Q8W: 66.6 (9.4) Fas 3 mg Q4W: 68.6 (7.6) Fas 6 mg Q8W: 65.2 (7.4);	67.1 (9.4)	Fas 1 mg Q4W: 27.0 (4.4) Fas 1 mg Q8W: 26.8 (4.2) Fas 3 mg Q4W: 24.8 (3.4) Fas 6 mg Q8W: 27.3 (4.5);	26.6 (4.3)	Fas 1 mg Q4W: 78.8% Fas 1 mg Q8W: 77.8% Fas 3 mg Q4W: 71.4% Fas 6 mg Q8W: 86.2%;	79.9%	Adults 40–85 year; ACR OA criteria; K–L ≥ 2; knee/hip OA	Fas 1 mg Q4W: Knee (190) or Hip (13) Fas 1 mg Q8W: Knee (150) or Hip (12) Fas 3 mg Q4W: Knee (26) or Hip (2) Fas 6 mg Q8W: Knee (26) or Hip (3) Placebo: Knee (189) or Hip (15)
NCT03691974* [31]	Fas: 62.9 (9.1)	62.4 (7.9)	NA	NA	Fas: 64.8%	70.8%	Adults ≥ 18 year; ACR OA criteria; K–L ≥ 2; knee/hip OA.	Knee or Hip

*RCT* Randomized Controlled Trial, *BMI* Body Mass Index (kg/m³), *Q4W* Every four weeks, *Q8W* Every eight weeks, *mg* Milligrams, *Fas* Fasinumab, *OA* Osteoarthritis, *K-L* Kellgren-Lawrence, *Age* Age in years, *ACR* American College of Rheumatology

*: All doses reported as administered Q4W (Every four weeks)

The average age of the included participants was 60.33 years with the maximum mean age of 68.60 years reported in the NCT03245008 trial [29]. Additionally, more than half of all participants were female, mean 70.61% (calculated as the unweighted average of the reported percentages across included study arms). The included studies population comprised individuals diagnosed with knee or hip OA, specifically those with a Kellgren-Lawrence (KL) grade equal to or greater than 2 [32].

### Risk of bias assessment

A risk of bias assessment was performed using the Cochrane Risk of Bias 2 (RoB 2) tool [22]. All included trials were judged to be at overall low risk of bias based on RoB 2 assessment. This judgement was based on adequate randomization, low risk of deviations from intended interventions, complete outcome data, appropriate outcome measurement, and absence of evidence of selective reporting [25–30]. Detailed domain-level assessments for each study are provided in Supplementary Fig. 1.

### Primary outcomes

**Fig. 2:**
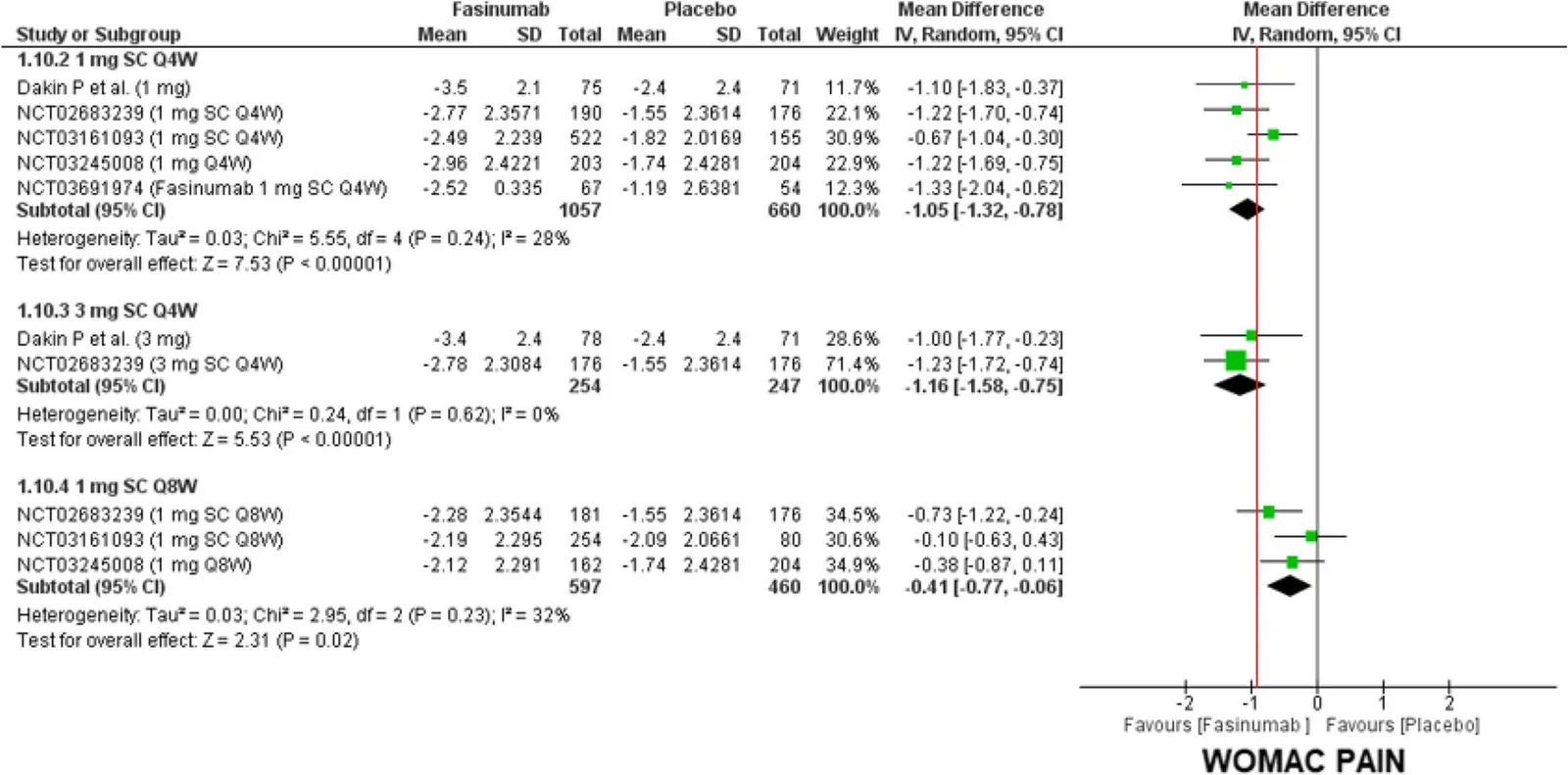
Forest plot of comparison: Fasinumab vs Placebo, outcome: WOMAC: Western Ontario and McMaster Universities Osteoarthritis Index; Pain at 16 weeks. SC: Subcutaneous; Q4W: Every Four Weeks; Q8W: Every Eight Weeks; mg: milligrams

WOMAC pain scores at 16 weeks were provided in five of the included studies for the 1 mg subcutaneous dose of Fasinumab and placebo [25, 26, 28–30]. The 1 mg Fasinumab administered every 4 weeks (1 mg SC Q4W) group had significantly lower pain scores than the placebo group [MD: -1.05, (95% C.I. -1.32 to -0.78), *p* < 0.00001, I²=28%]. Two studies investigated a 3 mg dose of Fasinumab every 4 weeks (3 mg SC Q4W) for this outcome and showed similar findings when compared with placebo [MD: -1.16, (95% C.I. -1.58 to -0.75), *p* < 0.00001, I²=0%] [25, 26]. Lastly, the group that was administered 1 mg Fasinumab every 8 weeks (1 mg SC Q8W) showed a smaller but significant mean difference in pain scores [MD: -0.41, (95% C.I. -0.77 to -0.06), *p* = 0.02, I²=32%] (Fig. 2). [26, 28, 29]. The 1 mg SC Q8W group showed significantly less WOMAC pain reduction than the 1 mg SC Q4W and 3 mg SC Q4W groups (*p* = 0.005 and *p* = 0.007, respectively). In comparison with the MCID (0.75–0.97), we found that the point estimates (MD) of the dosage regimen of 1 mg SC Q4W and 3 mg SC Q4W crossed the MCID values. However, the 95% confidence intervals extend to effects smaller than the conservative MCID threshold of 0.97, limiting certainty that the true effect reaches clinical significance. In contrast, the point estimate (MD) of 1 mg SC Q8W didn’t reach the MCID value. A Baujat plot is also shown in Supplementary Fig. 2.

**Fig. 3:**
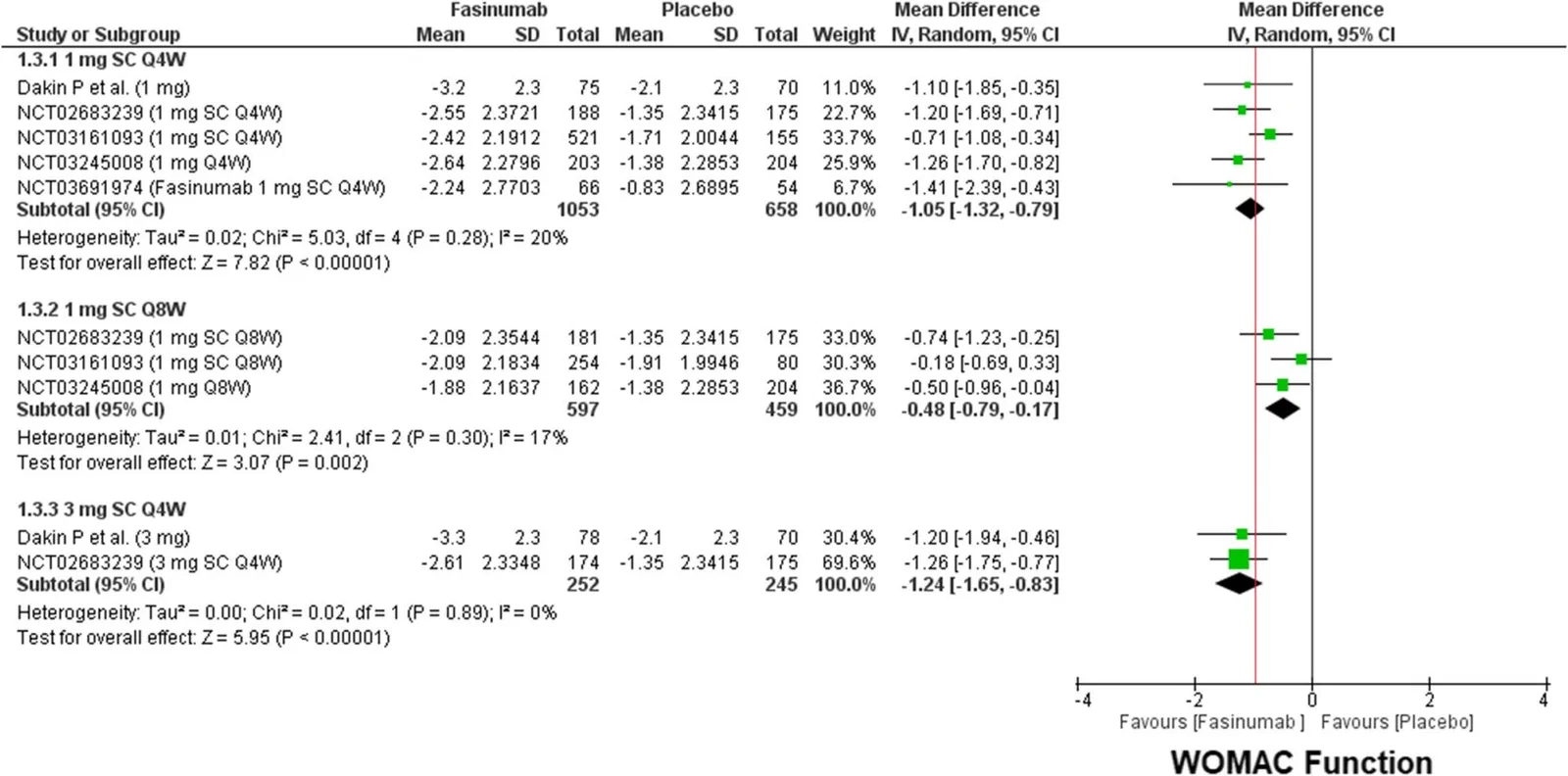
Forest plot of comparison: Fasinumab vs Placebo, outcome: WOMAC: Western Ontario and McMaster Universities Osteoarthritis Index; Function at 16 weeks. SC: Subcutaneous; Q4W: Every Four Weeks; Q8W: Every Eight Weeks; mg: milligrams

WOMAC function scores at 16 weeks were reported by 5 studies [25, 26, 28–30]. When 1 mg SC Q4W was used, it resulted in a MD of [-1.05 (95% C.I. -1.32 to -0.79), *p* < 0.00001, I²=20%] more than placebo. However, 1 mg SC Q8W also led to a significant but much smaller decrease in WOMAC function scores than the 1 mg SC Q4W group [MD: -0.48, (95% C.I. -0.79 to -0.17), *p* = 0.002, I²=17%]. Lastly, when the 3 mg SC Q4W drug regime was followed, it resulted in a decrease in WOMAC function scores by [MD: -1.24, (95% C.I. -1.65 to -0.83), *p* < 0.00001, I²=0%] more than placebo (Fig. 3). Like WOMAC pain, 1 mg SC Q8W provided significantly lower WOMAC function score reduction than the 1 mg SC Q4W and 3 mg SC Q4W groups (*p* = 0.006 and *p* = 0.004, respectively). Similar to WOMAC pain scores, when point estimates (MD) of WOMAC function scores were compared with the MCID (0.67–0.93), we found that point estimates of the dosage regimen of 1 mg SC Q4W and 3 mg SC Q4W exceeded the MCID values. However, the 95% confidence intervals include effects smaller than the conservative MCID threshold of 0.93, limiting certainty that the true effect reaches clinical significance. Further, the point estimates (MD) of the 1 mg SC Q8W dosage regimen didn’t reach the MCID values. A Baujat plot is also available in Supplementary Fig. 3.

**Fig. 4:**
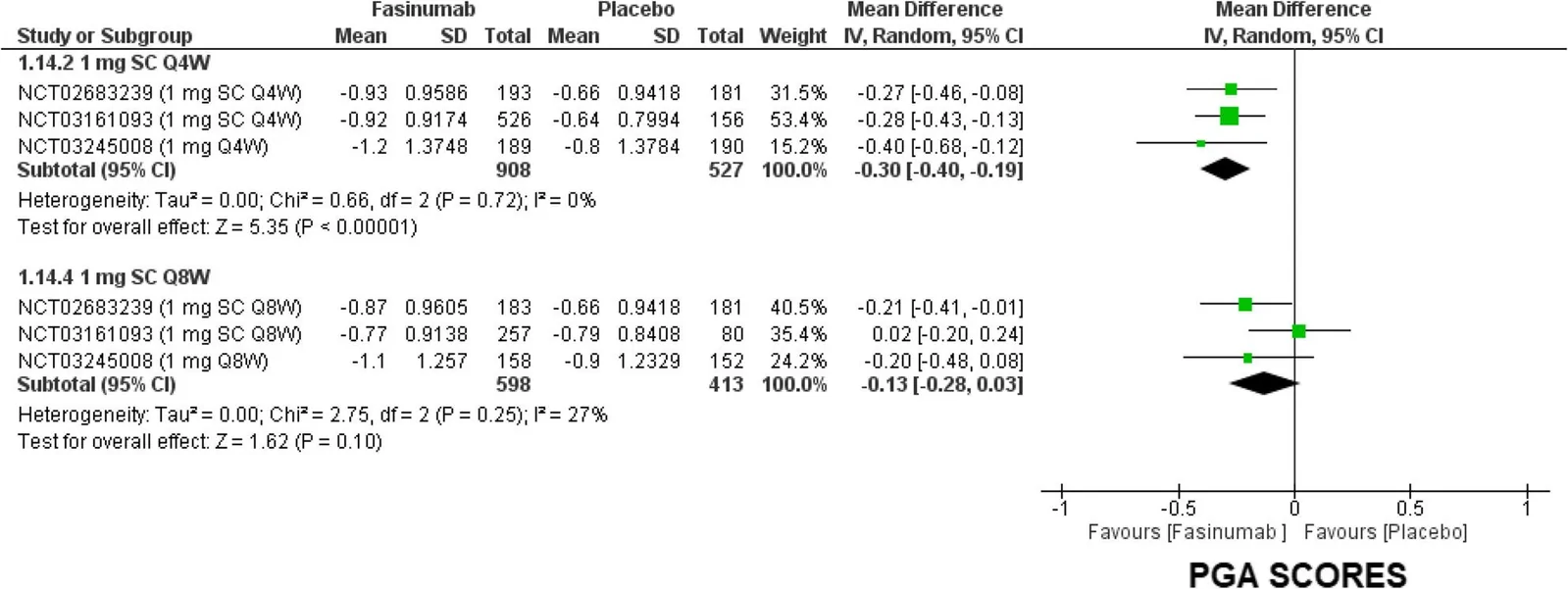
Forest plot of comparison: Fasinumab vs Placebo, outcome: PGA: Patient Global Assessment; at 16 weeks. SC: Subcutaneous; Q4W: Every Four Weeks; Q8W: Every Eight Weeks; mg: milligrams

The PGA at the 16-week mark was reported in four studies [25, 26, 28, 29]. However, one study [25] provided only p-values and did not report the numerical data required for quantitative synthesis. Consequently, this study was excluded from the meta-analysis, and the pooled PGA analysis included three studies [26, 28, 29]. When 1 mg SC Q4W was used, a significant improvement in PGA was observed compared with placebo. In contrast, the 1 mg SC Q8W regimen did not result in a statistically significant difference in PGA scores (Fig. 4).

A bar chart is available for a visual representation of the primary outcomes [Supplementary Figure 4].

### Secondary outcomes

All six RCTs reported patients who underwent Joint Replacement Surgery [25–30]. The need for joint replacement surgery after administering the drug in the 1 mg SC Q4W group was increased as compared to placebo. The lowest risk was observed in the 1 mg SC Q8W group compared to the placebo group. The 3 mg SC Q4W and 6 mg SC Q8W groups also had an increased risk of requiring joint replacement surgery compared to placebo [Supplementary Fig. 5]. The highest risk of all-cause joint replacement was found in the 6 mg SC Q8W and 3 mg SC Q4W categories.

The risk of adjudicated arthropathy was reported only for the 1 mg SC Q4W dosage regimen in five studies [25–28, 30]. Statistically significant evidence indicated that Fasinumab at this dosage was associated with an increased risk of adjudicated arthropathy compared to placebo. [Supplementary Fig. 6].

All six RCTs reported Adverse Events associated with the intervention and placebo [25–30]. The risk of adverse events in those receiving Fasinumab in the 1 mg SC Q4W and 1 mg SC Q8W categories was significantly higher than those receiving placebo. Conversely, there was no significantly increased risk of adverse events in the 3 mg SC Q4W group and the 6 mg SC Q8W groups [RR: 0.70, (95% C.I. 0.40 to 1.22), *p* = 0.21, I²=95%] [RR: 0.83, (95% C.I. 0.55 to 1.26), *p* = 0.38, I²=92%]; however the high heterogeneity of these two specific categories must be noted [Supplementary Fig. 7].

Leave-one-out sensitivity analysis identified NCT02683239 as an influential study in the 6 mg SC Q8W subgroup; after its exclusion, heterogeneity was reduced (I² = 0%), with a pooled estimate of [RR: 0.69, (95% CI 0.57 to 0.84); *p* = 0.0002]. 27] [Supplementary Fig. 8].

Serious Adverse Events were reported in five RCTs [26–30]. The 1 mg SC Q4W group had a significantly increased risk of serious adverse events. In contrast, neither the 1 mg SC Q8W, 3 mg SC Q4W, nor the 6 mg SC Q8W groups resulted in a significantly increased risk of serious adverse events [RR: 1.04, (95% C.I. 0.88 to 1.24), *p* = 0.63, I²=0%], [RR: 0.74, (95% C.I. 0.19 to 2.97), *p* = 0.67, I²=87%], and [RR: 0.71, (95% C.I. 0.15 to 3.35), *p* = 0.67, I²=88%], respectively; however, the high heterogeneity with the 3 mg SC Q4W, and the 6 mg SC Q8W groups should be noted [Supplementary Fig. 9]. Leave-one-out sensitivity analysis identified NCT02683239 as an influential study in the 3 mg SC Q4W and 6 mg SC Q8W subgroups; after its exclusion, heterogeneity was reduced (I² = 0%), with pooled estimates of [RR 0.36, (95% CI 0.17 to 0.75); *p* = 0.007] and [RR 0.32 (95% CI 0.15 to 0.71); *p* = 0.005], respectively [26]. [Supplementary Fig. 10].

All-cause mortality was reported in four RCTs [26–28, 30]. The analysis showed no significant increase in mortality risk with Fasinumab at dosage of 1 mg SC Q4W, 1 mg SC Q8W, 3 mg SC Q4W, and 6 mg SC Q8W [Supplementary Fig. 11].

Four RCTs reported on Sympathetic Nervous System Dysfunction [26–28, 30]. In the 1 mg SC Q4W regimen, there were no cases of sympathetic nervous system dysfunction in the Fasinumab group. However, one case of sympathetic nervous system dysfunction was observed in one of the placebo groups in one trial [28][Supplementary Fig. 12].

Three RCTs reported on peripheral sensory adverse events [26–28]. Peripheral sensory adverse events occurred significantly more in the 1 mg SC Q4W group than placebo.However, the number of peripheral sensory adverse events did not significantly differ between the treatment and placebo group in the 1 mg SC Q8W [Supplementary Fig. 13]. The high heterogeneity, however, should be noted.

Treatment-emergent adverse events were reported in five RCTs and were significantly greater in the intervention when 1 mg SC Q4W and the 6 mg SC Q8W regimens were used [26–30]. In contrast, treatment-emergent adverse events did not significantly differ between the placebo group and the 1 mg SC Q8W or 3 mg SC Q4W regimens[Supplementary Fig. 14].

Rapidly progressing osteoarthritis (RPOA) in patients receiving Fasinumab compared to placebo was reported in all six studies. The results demonstrate a statistically significant risk increase in RPOA with Fasinumab for for dosages 1 mg SC Q8W, 1 mg SC Q4W, 3 mg SC Q4W, 6 mg SC Q4W, 6 mg SC Q8W, and 9 mg SC Q4W[Supplementary Fig. 15].

Lastly, two studies reported on the risk of peripheral edema with Fasinumab treatment and placebo. Our analysis showed that none of the dosing regimens discovered an elevated risk of peripheral edema, and all 95% confidence intervals crossed the no effect line. [Supplementary Fig. 16].

The summary of these adverse secondary outcomes are condensed in Supplementary Table 3.

## Discussion

This meta-analysis of six RCTs involving 9,429 participants evaluated the safety and efficacy of Fasinumab across different dosing regimens. At 16 weeks, select regimens demonstrated statistically significant reductions in WOMAC pain, function, and PGA scores compared with placebo. However, when interpreted against conservative published MCID thresholds, the 95% confidence intervals include effects smaller than these thresholds, limiting certainty regarding clinically meaningful benefit. Furthermore, Fasinumab is associated with various adverse events.

While the impact of Fasinumab varies across different dosages and intervals, the most pronounced improvements in WOMAC pain and function scores were observed with a dosage of 3 mg administered every four weeks [Supplementary file 4] (Figure 2) (Figure 3). However, we didn’t find any statistical difference between the efficacy of 1 mg Fasinumab administered every four weeks and 3 mg Fasinumab administered every four weeks (*p* = 0.64) for WOMAC pain and (*p* = 0.45) for WOMAC function; this could either be due to limited power or that higher doses do not confer additional clinically meaningful benefit. Furthermore, PGA scores showed significant improvement compared to placebo, albeit limited to the 1 mg subcutaneous dose administered every four weeks. These results obtained at different dosages and different intervals were found to be variable to each other. Overall, the results suggest a possible trend toward greater efficacy at higher doses, but this pattern was not statistically confirmed across outcomes.Consequently, advancing treatment strategies for Fasinumab recipients necessitates the development and exploration of a broader spectrum of dosing regimens, ultimately aiming to maximize benefits for patients.

Over the last few years, there has been a debate about the fact statistical differences do not necessarily translate to clinically meaningful outcomes [33]. Consequently, the introduction of the concept of MCID has proven invaluable for clinicians in assessing therapeutic choices and determining whether significant outcomes hold genuine clinical relevance [34]. MCID helps clinicians evaluate therapeutic options by identifying the minimum change in an outcome measure that patients perceive as meaningful. Hence, we compared our WOMAC pain and WOMAC function scores with the MCID values reported in the literature to add another dimension to our results. Our results suggested that 1 mg SC Q8W may not provide any clinically meaningful effect. For the 1 mg SC Q4W and 3 mg SC Q4W regimens, point estimates exceeded conservative MCID thresholds; however, the 95% confidence intervals include effects smaller than the MCID threshold, indicating that the clinical relevance remains uncertain.

This further highlights the importance of a correct dosage regimen being prescribed to the patient as not all dosage regimens will likely prove beneficial to the patients.

Our results suggest that fasinumab is associated with increased risks of adverse outcomes, including joint replacement surgery, adjudicated arthropathy, peripheral sensory adverse events, RPOA, and TEAEs. However, we suggest that these health detriments may be more prominent at higher or more frequent Fasinumab drug regimens; as seen by our results with the need for Joint Replacement. This again highlights the importance and need for further research into the safety and side effects of this drug and the risk-to-benefit ratio associated with different drug dosages of Fasinumab. Until more robust long-term data are available, clinicians should refrain from using fasinumab as first-line therapy for osteoarthritis [35].

The dose-related increase in adverse events associated with Fasinumab is most likely due to the central mechanism of NGF inhibition.

One proposed mechanism is that reduced pain sensation following inhibition of NGF might increase joint loading and overuse, leading to accelerated deterioration in the joint structure [36]. There has also been a hypothesis that NGF inhibition might affect neuroimmune communication, angiogenesis, and tissue repair, which might lead to injury to the subchondral bone and degradation of the articular cartilage [37]. Yet, it must be noted that these mechanisms still remain unclear.

These mechanistic findings are consistent with our results that increased doses (e.g., 3 mg Q4W and 6 mg Q8W) were linked with significantly increased risks of RPOA and joint replacement rates [38].

When comparing our findings with other studies and anti-NGF agents, a meta-analysis by Schnitzer et al. demonstrated favorable outcomes with both fulranumab and fasinumab, although dose-specific stratifications were not performed for these agents due to limited data availability. Schnitzer et al. demonstrated favorable outcomes with tanezumab, fulranumab, and fasinumab, reporting improvements in WOMAC pain and function outcomes, although dose-specific analyses for fulranumab and fasinumab were limited by available data [39]. While their use of standardized mean differences limits direct comparison with our mean difference estimates, the overall direction of effect supports the efficacy of NGF inhibition. Regarding safety, lower doses of tanezumab (2.5 mg and 5 mg) were not associated with increased withdrawals due to adverse events, whereas the 10 mg dose demonstrated a significantly higher riskFor fulranumab, the estimated odds ratio for withdrawals due to AEs was 1.77 (0.74–4.22), also not statistically different from placebo, though interpretation was limited by small sample sizes and low event rates. Evaluation of fasinumab safety was also limited in that analysis due to only one available study with few events.

Our study complements earlier work by providing updated evidence on Fasinumab, particularly regarding its effects on WOMAC pain and function scores at 16 weeks. Similar to observations with Tanezumab, point estimates for higher Fasinumab doses were numerically greater, though confidence intervals indicate uncertainty regarding additional clinical benefit. Schnitzer et al. had also concluded that the benefits of NGF inhibitors may be class-wide but noted a lack of data on fasinumab. Our analysis addresses this gap by showing that it is not certain if fasinumab may offer clinically meaningful short-term symptom relief. As also noted in their prior work on tanezumab, our results suggest a dose-response trend, visible in few of our forest plots. It is also worth noting that, although not explicitly stated, the trials included in these analyses are largely of short-term duration (< 32 weeks). Therefore, the long-term efficacy and safety profile of these agents remains uncertain [39].

Similarly, another meta-analysis focusing on Tanezumab found that the intervention helps more than a placebo in alleviating WOMAC pain scores of OA patients [40]. Consistent with the prior meta-analysis and our results, their subgroup also demonstrated that the effect rises in higher doses. Similar results were also reported for the WOMAC function scores and PGA scores. For safety exploration, the authors found that tanezumab increases the risk of RPOA; however, SAE and Joint replacement events were not statistically significant compared to placebo, although it is to be noted that they didn’t stratify their results for the SAE and Joint replacement plots by dosages. However, a different meta-analysis, which stratified their results for these outcomes, also found no significant difference between Tanezumab and Placebo, suggesting that, unlike Fasinumab based on our results, Tanezumab might not have adverse events related to Joint replacement events and Serious Adverse Events [41]. Although, they did report that Tanezumab might be associated with abnormal peripheral sensation. Our results with Fasinumab (Peripheral sensory adverse events) also revealed such association.

Regarding fulranumab, one meta-analysis has provided insight into its short-term safety and efficacy. Similar to tanezumab, fulranumab demonstrated positive outcomes. Interestingly, unlike fasinumab and tanezumab, the analysis did not identify any statistically significant increase in adverse events or SAEs associated with fulranumab. Further investigations are needed into this as the meta-analysis didn’t have a widespread exploration on the drug’s safety profile, for example risk of RPOA [42].

According to the ACR guidelines, the use of anti-NGF agents is not recommended as first-line routine therapy for osteoarthritis due to concerns regarding safety and lack of information on long-term outcomes [43]. In addition, fasinumab has not been approved by any regulatory authority [25]. Development of anti-NGF therapies has faced many hurdles in terms of safety concerns that include rapidly progressive osteoarthritis and dose-related arthropathies, observed in clinical trials. Moreover, despite showing promise in alleviating pain in patients, the potential large cost involved in their use might pose challenges in their widespread application, considering that their cost-effectiveness compared with traditional medications like NSAIDs or corticosteroids remains unknown.

Compared with conventional OA therapies such as NSAIDs, corticosteroids, opioids, and COX-2 inhibitors, anti-NGF agents have demonstrated the greatest effect sizes for pain reduction and functional improvement in network meta-analysis of 38 RCTs involving over 20,000 patients [44]. However, despite their superior efficacy, anti-NGF therapies may carry greater safety concerns due to structural adverse effects, including arthropathy, compared with traditional therapies. While NSAIDs are associated with gastrointestinal and cardiovascular risks and corticosteroids with long-term complications, their safety profiles are better established and more predictable [44]. Therefore, agents such as fasinumab may be better reserved as second-line therapies for patients unresponsive to conventional treatments, with careful monitoring for joint deterioration, particularly in high-risk populations.

As previously highlighted, the lack of clarity regarding its potential costs versus the existing medications for osteoarthritis poses yet another hurdle for the use of Fasinumab. Due to its high price in comparison with conventional therapies along with absence of its cost-effectiveness data, its clinical applicability remains obscure [45].

Another obstruction is our limited understanding of how well Fasinumab works over the long haul. To the best of our knowledge, no study exists that evaluates long term efficacy (> 1 year) of Fasinumab. Osteoarthritis is a chronic ailment, and outcomesat 16 weeks may not be justifiable for its use. Hence, Fasinumab’s long term effectiveness is another significant gap in our knowledge which future studies must address.

Like any other study, this study also has its limitations. First, the sensitivity of certain AE and SAE subgroup analyses to exclusion of a single study indicates that these findings may be influenced by study-level characteristics. Therefore, safety outcomes in the 3 mg SC Q4W and 6 mg SC Q8W regimens should be interpreted with caution [26]. Few of our secondary outcomes also reported high heterogeneity. Potential sources of this heterogenity could be due to the variation in time when the outcomes were reported or the differences in treatment periods of individual studies. It could also be due to the range of KL grades (II-IV) employed in our included studies. Further, few safety outcomes were based on small event numbers, reflected in wide confidence intervals, and should therefore be interpreted cautiously. Another limitation of this meta-analysis is the wide range in the age groups in most of the studies included in the meta-analysis. It has been noticed quite frequently that a drug effective in one age group is not as effective in the other [46].

Another key issue is although baseline Kellgren–Lawrence (KL) grades were recorded ( > = 2), further subgroup analyses based on radiographic severity were not reported in trials for us to analyze for example outcomes in KL grade 3 patients versus KL grade 4 patients. Prior evidence suggests that patients with KL grades 3–4 are at higher risk of progression and may thus experience higher rates of joint replacement on Fasinumab [47]. Furthermore, outcomes were not stratified in the trials based on anatomical joint location, hip, or knee, preventing evaluation on the difference in safety and efficacy between joint types. Lastly, an additional limitation relates to the interpretation of MCID thresholds. While an MCID value was reported in one of the included Fasinumab trials [25], we applied a more conservative threshold provided in the Tanezumab meta-analysis to avoid overestimating clinical benefit [24]. The interpretation of clinical meaningfulness may differ if alternative thresholds are applied. Therefore, conclusions regarding clinical meaningfulness should be interpreted within this context.

Regardless of the limitations, this meta-analysis is the first meta-analysis that thoroughly evaluates the efficacy and safety of Fasinumab over placebo.

## Conclusion

Although select Fasinumab regimens demonstrated statistically significant reductions in WOMAC pain, function, and PGA scores at 16 weeks, confidence interval overlaps with conservative MCID thresholds limit certainty regarding clinically meaningful efficacy. Additionally, treatment may carry increased risks, including adjudicated arthropathy and joint replacement surgeries. Further research is needed to confirm these findings.

## Supplementary Information

Supplementary Material 1.

## Data Availability

Data extracted from the included published studies and the dataset generated for the meta-analyses are available from the corresponding author upon reasonable request.

## Abbreviations

OA: Osteoarthritis
RCTs: Randomized control trials
BMI: Body mass index
WOMAC: Western Ontario and McMaster Universities Osteoarthritis Index
PGA: Patient Global Assessment
TEAEs: Treatment Emergent Adverse Events
RPOA: Rapidly Progressing Osteoarthritis
RR: Risk Ratio
MCID: Minimal clinically important difference
PROM: Patient Reported Outcome Measure
WHO: World Health Organization
KL: Kellgren-Lawrence
RoB 2: Risk of Bias 2
SC: Subcutaneous
MD: Median dose
SMD: Standardized mean difference
