# Supplementary material for: Fasinumab for osteoarthritis: a systematic review and meta-analysis of efficacy and safety
Source: BMC Musculoskelet Disord. 2026 Jul 24;27:888. doi: 10.1186/s12891-026-10271-8 (PMC13681872; doi:10.1186/s12891-026-10271-8)
Supplement: Supplementary file 1 [file 12891_2026_10271_MOESM1_ESM.docx]

Supplementary files for

**Fasinumab for Osteoarthritis: A Systematic Review and Meta-Analysis of Efficacy and Safety**

**Author Information:**

1. Muhammad Sohaib Khan
2. Wania Moeen
3. Ummesalma Shabbar Banatwala
4. Muhammad Usman
5. Umar Mahmood
6. Syed Ashad Ahmed Fatmi
7. Syed Muhammad Muneeb Akhtar
8. Ashir Kanwal
9. Hania Shahzad, MD
10. Muhammad Talal Ibrahim, MD
11. Prof. Badaruddin Sahito, MBBS, FCPS, FRCSEd (Tr & Orth)
12. Muhammad Sohaib Asghar **[Corresponding Author]**

Affiliation : AdventHealth Sebring, FL, USA
Email : msohaibasghar123@gmail.com

**Supplementary Table 1. Details of Search Strategy**

| **Databases** | **Search Strategy** |
| --- | --- |
| Pubmed | (REGN475 OR "REGN 475" OR REGN-475 OR Fasinumab OR MT5547 OR MT-5547) AND ("Osteoarthritis"[MeSH Terms] OR osteoarthritis OR "osteo-arthritis" OR OA) |
| Cochrane | (REGN 475 OR Fasinumab OR MT-5547 OR REGN475 OR REGN 475 OR MT5547 OR MT 5547) AND (Osteoarthritis OR OA) |
| Clinicaltrials.gov | Condition/disease = Osteoarthritis Intervention/Treatment= (REGN 475 OR Fasinumab OR MT-5547) |
| Google Scholar | (REGN 475 OR Fasinumab OR MT-5547 OR REGN475 OR REGN-475 OR MT 5547) AND Osteoarthritis |

**Supplementary Table 2. Definitions of Outcomes**

| **Outcome** | **Definition** |
| --- | --- |
| Adverse Event | Any unexpected medical incident in a participant/patient who has been given a study drug that may or may not have a causal relationship with the study drug |
| Serious Adverse Event | Any untoward medical incident that resulted in any of the following outcomes: death, life-threatening, required initial or prolonged in-patient hospitalization, persistent or significant disability/incapacity, congenital anomaly/birth defect, or considered as a medically important event. |
| Peripheral Sensory Adverse Event | Defined as any peripheral sensory adverse event that required a neurology consultation. |
| Treatment Emergent Adverse Events | Defined as an Adverse Event with an onset that occurs after receiving a study drug |
| Adjudicated Arthropathy | Adjudicated Arthropathy is a composite term that encompass: Rapidly progressive Osteoarthritis type 1 and 2, Subchondral insufficiency fractures, and Primary Osteonecrosis. |
| Patient Global Assessment | The Patient Global Assessment of Osteoarthritis is a patient-rated assessment of current disease state on a 5-point Likert scale. Lower numbers indicate better patient conditions. (1 = very good; 2 = good; 3 = fair; 4 = poor; and 5 = very poor). |
| Western Ontario and McMaster Universities Osteoarthritis Index (WOMAC) | The WOMAC index consists of 24 parameters grouped in 3 subscales (pain- contains 5 questions, physical function contains 17 questions and stiffness consists of 2 questions), with 0-10 grading of each question. The scores for each subscale are summed up, divided by number of questions, and each subscale is reported using Numerical Rating Scale score of 0-10. Higher scores suggests worse pain, functional and stiffness limitations. |
| Sympathetic Nervous System Dysfunction | Abnormalities or dysregulation in the sympathetic branch of the autonomic nervous system. Events related to sympathetic nervous system dysfunction were monitored through physical examination, Adverse Events reporting, assessment of orthostatic hypotension, and the Survey of Autonomic Symptoms. |

**Supplementary Figure 1.** Risk of Bias assessment for included Randomized Controlled Trials (RCTs).

**Supplementary Figure 2.** Baujat plot for evaluating heterogeneity patterns in meta-analysis. The Baujat plot illustrates the interplay between effect size influence (y-axis) and heterogeneity contribution (x-axis), aiding in the identification of influential cases and potential outliers. SC: Subcutaneous; Q4W: Every Four Weeks; Q8W: Every Eight Weeks; mg: milligrams.

**Supplementary Figure 3.** Baujat plot for evaluating heterogeneity patterns in meta-analysis. The Baujat plot illustrates the interplay between effect size influence (y-axis) and heterogeneity contribution (x-axis), aiding in the identification of influential cases and potential outliers. SC: Subcutaneous; Q4W: Every Four Weeks; Q8W: Every Eight Weeks; mg: milligrams.

**Supplementary Figure 4.**  Bar-chart displaying results of pooled proportion of variables. SC: Subcutaneous; Q4W: Every Four Weeks; Q8W: Every Eight Weeks; mg: milligrams; WOMAC: Western Ontario and McMaster Universities Osteoarthritis Index.

**Supplementary Figure 5.**  Forest plot of comparison: Fasinumab vs Placebo, outcome: **All-Cause Joint Replacement Surgery.** Risk ratios below 1 favor Fasinumab (fewer adverse events in Fasinumab). SC: Subcutaneous; Q4W: Every Four Weeks; Q8W: Every Eight Weeks; mg: milligrams.

**Supplementary Figure 6.** Forest plot of comparison: Fasinumab vs Placebo, outcome: **Adjudicated Arthropathy**. Risk ratios below 1 favor Fasinumab (fewer adverse events in Fasinumab). SC: Subcutaneous; Q4W: Every Four Weeks; mg: milligrams.

**Supplementary Figure 7.** Forest plot of comparison: Fasinumab vs Placebo, outcome: **Adverse Events.** Risk ratios below 1 favor Fasinumab (fewer adverse events in Fasinumab). SC: Subcutaneous; Q4W: Every Four Weeks; Q8W: Every Eight Weeks; mg: milligrams

**Supplementary Figure 8.** Forest plot of comparison: Fasinumab vs Placebo, outcome: **Adverse Events (After leave one out analysis)**. Risk ratios below 1 favor Fasinumab (fewer adverse events in Fasinumab). SC: Subcutaneous; Q4W: Every Four Weeks; Q8W: Every Eight Weeks; mg: milligrams.

**Supplementary Figure 9.** Forest plot of comparison: Fasinumab vs Placebo, outcome: **Serious Adverse Events**. Risk ratios below 1 favor Fasinumab (fewer adverse events in Fasinumab). SC: Subcutaneous; Q4W: Every Four Weeks; Q8W: Every Eight Weeks; mg: milligrams.

**Supplementary Figure 10.** Forest plot of comparison: Fasinumab vs Placebo, outcome: **Serious Adverse Events (After leave one out analysis)**. Risk ratios below 1 favor Fasinumab (fewer adverse events in Fasinumab). SC: Subcutaneous; Q4W: Every Four Weeks; Q8W: Every Eight Weeks; mg: milligrams.

**Supplementary Figure 11.** Forest plot of comparison: Fasinumab vs Placebo, outcome: **All cause mortality**. Risk ratios below 1 favor Fasinumab (fewer adverse events in Fasinumab). SC: Subcutaneous; Q4W: Every Four Weeks; Q8W: Every Eight Weeks; mg: milligrams.

**Supplementary Figure 12.** Forest plot of comparison: Fasinumab vs Placebo, outcome: **Sympathetic Nervous System Dysfunction.** Risk ratios below 1 favor Fasinumab (fewer adverse events in Fasinumab). SC: Subcutaneous; Q4W: Every Four Weeks; Q8W: Every Eight Weeks; mg: milligrams.

**Supplementary Figure 13.** Forest plot of comparison: Fasinumab vs Placebo, outcome: **Peripheral Sensory Adverse Events.** Risk ratios below 1 favor Fasinumab (fewer adverse events in Fasinumab). SC: Subcutaneous; Q4W: Every Four Weeks; Q8W: Every Eight Weeks; mg: milligrams.

**Supplementary Figure 14.** Forest plot of comparison: Fasinumab vs Placebo, outcome: **Treatment Emergent Adverse Events.** Risk ratios below 1 favor Fasinumab (fewer adverse events in Fasinumab). SC: Subcutaneous; Q4W: Every Four Weeks; Q8W: Every Eight Weeks; mg: milligrams. (xx1093 has in events so excluded)

**Supplementary Figure 15.** Forest plot of comparison: Fasinumab vs Placebo, outcome: **Rapidly Progressing Osteoarthritis.** Risk ratios below 1 favor Fasinumab (fewer adverse events in Fasinumab). SC: Subcutaneous; Q4W: Every Four Weeks; Q8W: Every Eight Weeks; mg: milligrams.

**Supplementary Figure 16.** Forest plot of comparison: Fasinumab vs Placebo, outcome: **Peripheral Edema**. Risk ratios below 1 favor Fasinumab (fewer adverse events in Fasinumab). SC: Subcutaneous; Q4W: Every Four Weeks; Q8W: Every Eight Weeks; mg: milligrams.

| Outcome | Dose | Risk Ratio (95%CI) | Significance (P-value) | Comments |
| --- | --- | --- | --- | --- |
| Joint replacement surgery | 1 mg SC Q4W | RR: 1.64, (95% C.I. 1.25 to 2.16) | p=0.0004 | Significant |
|  | 1 mg SC Q8W | RR: 1.39, (95% C.I. 1.02 to 1.89) | p=0.04 | Significant |
|  | 3 mg SC Q4W | RR: 2.42, (95% C.I. 1.54 to 3.82) | p=0.0001 | Significant |
|  | 6 mg SC Q8W | RR: 2.65, (95% C.I. 1.99 to 3.54) | p<0.00001 | Significant |
| Risk of adjudicated arthropathy | 1 mg SC Q4W | RR: 3.94, (95% C.I. 2.82 to 5.51) | p<0.00001 | Significant |
| Adverse Events | 1 mg SC Q4W | RR: 1.08, (95% C.I. 1.04 to 1.12 | p<0.00001 | Significant |
|  | 1 mg SC Q8W | RR: 1.06, (95% C.I. 1.01 to 1.11) | p=0.03 | Significant |
|  | 3mg SC Q4W | RR: 0.70, (95% C.I. 0.40 to 1.22) | p=0.21 | Not significant |
|  | 6 mg SC Q8W | RR: 0.83, (95% C.I. 0.55 to 1.26) | p=0.38 | Not significant |
|  | 6 mg SC Q8W | RR: 0.69, (95% CI 0.57 to 0.84) | p = 0.0002 | Significant |
| Serious Adverse Events | 1 mg SC Q4W | RR: 1.23, (95% C.I. 1.06 to 1.43) | p=0.0006 | Significant |
|  | 1 mg SC Q8W | RR: 1.04, (95% C.I. 0.88 to 1.24) | p=0.63 | Not significant |
|  | 3 mg SC Q4W | RR: 0.74, (95% C.I. 0.19 to 2.97 | p=0.67 | Not significant |
|  | 6 mg SC Q8W | RR: 0.71, (95% C.I. 0.15 to 3.35) | p=0.67 | Not significant |
|  | 3 mg SC Q4W | RR 0.36, (95% CI 0.17 to 0.75) | p = 0.007 | Significant |
|  | 6 mg SC Q8W | RR 0.32 (95% CI 0.15 to 0.71) | p = 0.005 | Significant |
| Mortality risk | 1 mg SC Q4W | RR: 0.97 (95% CI: 0.49–1.90) | p=0.93 | Not significant |
|  | 1 mg SC Q8W | RR: 1.07 (95% CI: 0.51–2.27) | p=0.86 | Not significant |
|  | 3 mg SC Q4W | RR: 0.26 (95% CI: 0.03–1.98) | p=0.19 | Not significant |
|  | 6 mg SC Q8W | RR: 0.89 (95% CI: 0.39–2.02) | p=0.77 | Not significant |
| Peripheral sensory adverse events | 1 mg SC Q4W | RR: 1.94, (95% C.I. 1.18 to 3.19) | p=0.009 | Significant |
|  | 1 mg SC Q8W group | RR: 1.76, (95% C.I. 0.63 to 4.91) | p=0.28 | Not significant |
| Treatment-emergent adverse events | 1 mg SC Q4W | RR: 1.09, (95% C.I. 1.05 to 1.12) | p<0.00001 | Significant |
|  | 6 mg SC Q8W | RR: 1.06, (95% C.I. 1.03 to 1.09) | p=0.0003 | Significant |
|  | 1 mg SC Q8W | RR: 1.24, (95% C.I. 0.73 to 2.11) | p=0.42 | Not significant |
|  | 3 mg SC Q4W | RR: 1.03, (95% C.I. 0.97 to 1.09) | p=0.33 | Not significant |
| Rapidly progressing osteoarthritis (RPOA) | 1 mg SC Q8W | RR: 4.16, (95% CI: 1.98 to 8.76) | p = 0.0002 | Significant |
|  | 1 mg SC Q4W | RR: 4.40, (95% CI: 2.37 to 8.15) | p < 0.00001 | Significant |
|  | 3 mg SC Q4W | RR: 6.24, (95% CI: 2.89 to 13.46) | p < 0.00001 | Significant |
|  | 6 mg SC Q4W | RR: 10.81, (95% CI: 4.34 to 26.92) | p < 0.00001 | Significant |
|  | 6 mg SC Q8W | RR: 10.42, (95% CI: 4.36 to 24.89) | p < 0.00001 | Significant |
|  | 9 mg SC Q4W | RR: 8.79, (95% CI: 3.31 to 23.37) | p < 0.00001 | Significant |
| Risk of peripheral edema | 1 mg SC Q8W | RR: 0.26, (95% CI: 0.01 to 5.41) | p = 0.38 | Not significant |
|  | 1 mg SC Q4W | RR: 0.49, (95% CI: 0.05 to 4.44) | p = 0.53 | Not significant |
|  | 3 mg SC Q4W | RR: 1.17, (95% CI: 0.06 to 24.29) | p = 0.92 | Not significant |
|  | 6 mg SC Q8W | RR: 0.15, (95% CI: 0.01 to 3.12) | p = 0.22 | Not significant |

**Supplementary Table 3.**
